# Complete chloroplast genome of a traditional medicinal plant *Luisia hancockii* Rolfe 1896: genomic features and phylogenetic relationship within subtribe Aeridinae (Orchidaceae)

**DOI:** 10.1080/23802359.2023.2275334

**Published:** 2023-10-30

**Authors:** Leqin Huang, Zhenyu Lu, Junfeng Wang, Huijuan Zhang, Ming Jiang

**Affiliations:** a College of Life Sciences, Taizhou University, Taizhou, China; b Scientific Research Management Center, East China Medicinal Botanical Garden, Lishui, China

**Keywords:** *Luisia hancockii*, plastid genome, phylogenetic analysis

## Abstract

*Luisia hancockii* Rolfe 1896 is an epiphytic orchid species. In our present study, the whole chloroplast genome sequence of *L. hancockii* was *de novo* assembled by using high-throughput Illumina reads, and phylogenetic analysis was conducted within species of subtribe Aeridinae. The complete chloroplast genome sequence of *L. hancockii* was 146,243 bp in length, with a typical quadripartite structure, and its large single-copy, small single-copy, and inverted repeat were 84,441 bp, 11,412 bp, and 25,195 bp long, respectively. The GC content of the whole chloroplast genome was 36.6%, while the GC contents of LSC, SSC, and IR were 33.8%, 27.5%, and 43.3%, respectively. The chloroplast genome consisted of 129 genes, including 74 protein-coding genes, eight rRNAs, 38 tRNAs, and nine pseudogenes. Phylogenic tree was generated using the best model GTR + R, and the results showed that *L. hancockii* was sister to *Holcoglossum* and *Vanda* species, with a support of 100%.

## Introduction

Orchidaceae is the second largest family in angiosperm, which consists of approximately 800 genera and over 25,000 species (Chase et al. 2015; Phillips et al. 2020). In China, there are more than 200 genera and over 1600 species, with 682 endemic to China (Zhou et al. 2016). *Luisia* is a small genus in Orchidaceae, and it is comprised of about 40 species in the world, and they are distributed in tropical and subtropical Asia, such as Bhutan, China, and India (Wu et al. 2009; Karuppusamy and Ravichandran 2019). About 11 *Luisia* species are distributed in China, and five are endemic. They are all herbs, epiphytic or lithophytic (Wu et al. 2009). *L. hancockii* is a perennial herb with tufted stems, obtuse leaves, and floral bracts broadly ovate, and it is often found on boulders, in rock crevices, or on tree trunks of *Cinnamomum camphora* (L.) Presl 1753, *Castanopsis sclerophylla* (Lindl. & Paxton) Schottky 1912, and *Pterocarya stenoptera* C. DC. 1862, according to our field investigation. *L. hancockii* is a medicinal plant that is commonly used to treat rheumarthritis, carbuncles, and laryngitis in folk medicine (Yao and Xiong 2016). To explore the phylogenomic relationship with other plants in subtribe Aeridinae (Orchidaceae), the chloroplast genome of *L. hancockii* was assembled, and a phylogenetic tree was generated. Our objectives were to investigate the features of *L. hancockii* chloroplast genome and to elucidate its phylogenetic relationship.

## Materials and methods

### Plant sampling

**Figure 1.**
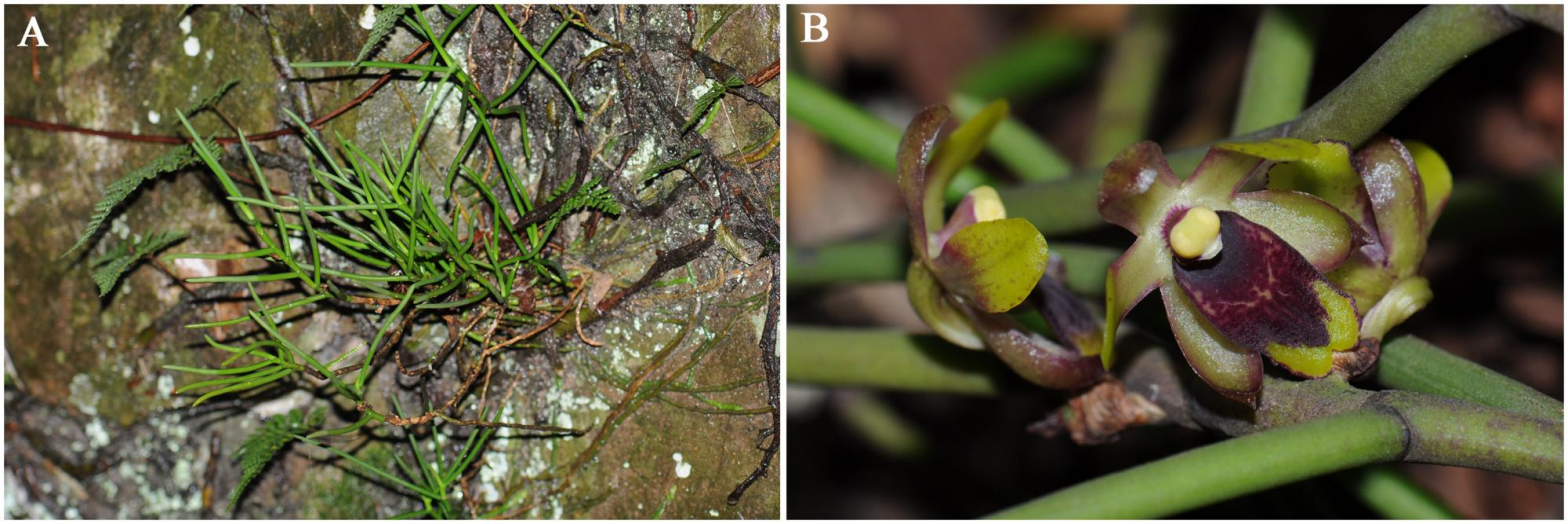
*Luisia hancockii* Rolfe 1896. (A) Habitat; (B) Flowers. Both photos were taken by Ming Jiang. *L. hancockii* is a perennial herb with a height of 10–20 cm. The flowers are fleshy, with sepals and petals in yellow-green color. The lip is nearly ovate-oblong, and lip hypochile is purple-red. The flowering period is from May to June.

Leaf samples were harvested from Huayan Mountain (28°41′24″N, 121°06′51″E), Huangyan District, Taizhou City, Zhejiang Province, China (Figure 1). A voucher specimen assigned CHS20180244 is deposited in Molecular Biology Innovation Laboratory at Taizhou University (Ming Jiang, jiangming1973@139.com).

### DNA isolation, sequencing, assembling, and annotation of the chloroplast genome

The CTAB (cetyltrimethylammonium bromide) method was used for DNA extraction (Doyle and Doyle 1987). A genomic DNA library was then constructed and sequenced using an Illumina Hiseq X Ten sequencing platform. Clean reads were obtained by filtering low-quality raw data reads with a Perl-based stand-alone program package NGSQCToolkit v2.3.3 (Patel and Jain 2012). As a result, a total of 11,362,518 high quality clean reads were generated, representing 3.41 G bases of nucleotide sequences. The whole chloroplast genome was *de novo* assembled by a Perl-based program NOVOPlasty (Dierckxsens et al. 2017). Annotation of the plastid genome was conducted using the Dual Organellar GenoMe Annotator (Wyman et al. 2004) combined with manual correction. tRNAscan-SE 2.0 was employed to predict tRNA, and RNAmmer was applied to identify rRNA genes (Lagesen et al. 2007; Chan and Lowe 2019). The whole chloroplast genome map of *L. hancockii* was drawn using CPGview (http://www.1kmpg.cn/cpgview/) (Liu et al. 2023).

### Phylogenetic analysis

To determine the phylogenetic position of *L. hancockii* within subtribe Aeridinae (Orchidaceae), 17 chloroplast genome sequences downloaded from NCBI, along with a sequence of *Cymbidium erythraeum* Lindl. 1858 (MK820373) as the outgroup. Multiple alignments were then performed with MAFFT v7.450 (Katoh and Standley 2013). A phylogenetic tree was created using whole plastome sequences based on maximum-likelihood method by PhyML 3.1 (Guindon et al. 2010), under the best-fit substitution model GTR + R.

## Results

**Figure 2.**
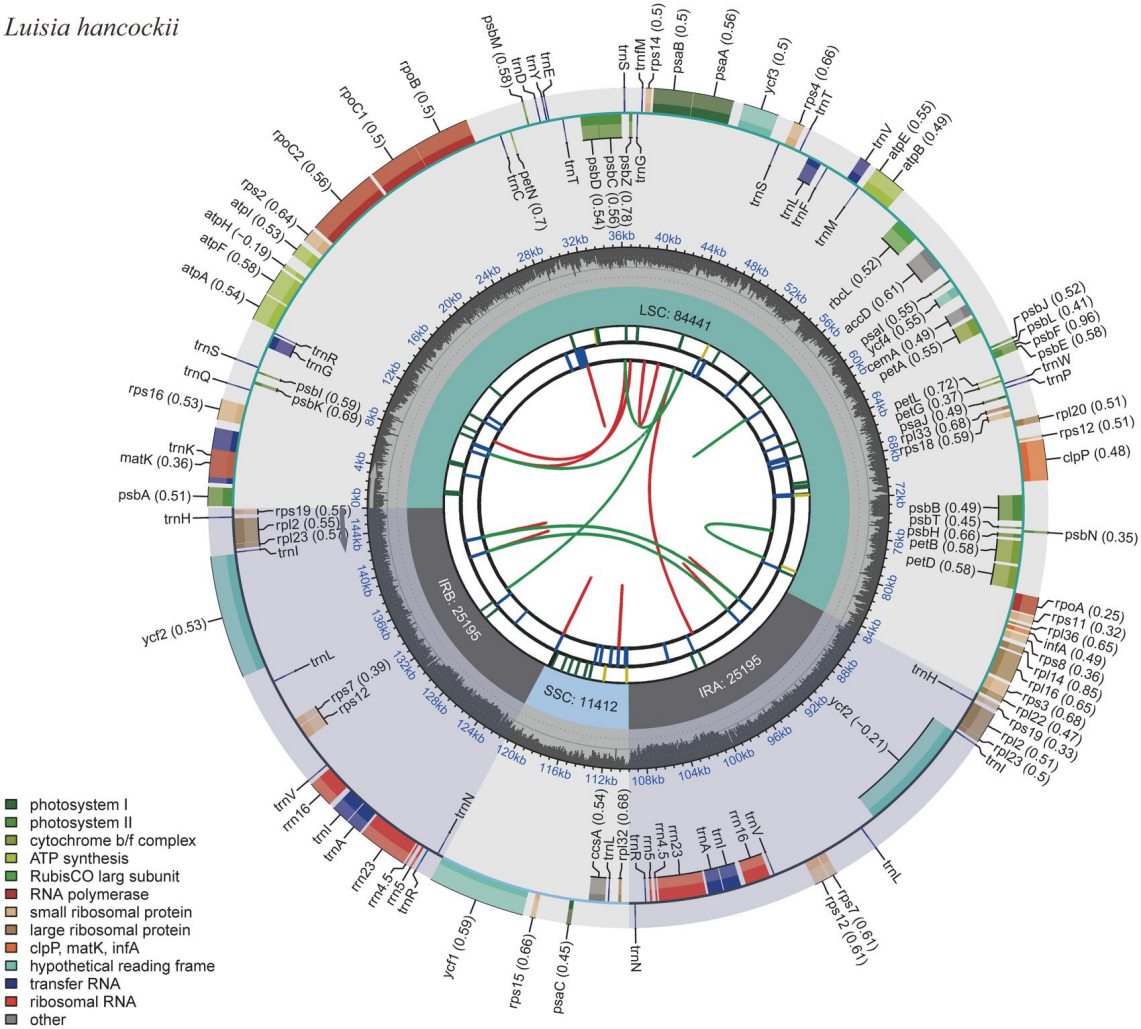
The chloroplast genome map of *Luisia hancockii*. The map contains six tracks. From the center outward, the first track shows the dispersed repeats, which consist of direct and palindromic repeats, connected with red and green arcs. The second track indicates the long tandem repeats as short blue bars. The third track reveals the short tandem repeats or microsatellite sequences as short bars with different colors. The colors, type of repeat they represent, and the description of the repeat types are as follows: black: c (complex repeat); green: p1 (repeat unit size = 1); yellow: p2 (repeat unit size = 2); purple: p3 (repeat unit size = 3); blue: p4 (repeat unit size = 4); orange: p5 (repeat unit size = 5); red: p6 (repeat unit size = 6). The chloroplast genome contains an LSC region, an SSC region, and two IR regions, and they are shown on the fourth track. The GC content along the genome is shown on the fifth track. Genes are color-coded according to their functional classification. The transcription directions for the inner and outer genes are clockwise and anticlockwise, respectively. The bottom left corner indicates the key for the functional classification of the genes.

The results revealed that the total length of the *Luisia hancockii* chloroplast genome covers 146,243 bp, exhibiting a typical circular quadripartite structure, with an average depth of ×460.68 (Figure S1). Its large single-copy (LSC), small single-copy (SSC), and inverted repeats (IRA and IRB) were 84,441 bp, 11,412 bp, and 25,195 bp long, respectively (Figure 2). The GC content of the whole plastome was 36.6%, while those of LSC, SSC, and IRs were 33.8%, 27.5%, and 43.3%, respectively. The chloroplast genome sequence of *L. hancockii* is released and available now in GenBank under an accession number of OR030420.

**Figure 3.**
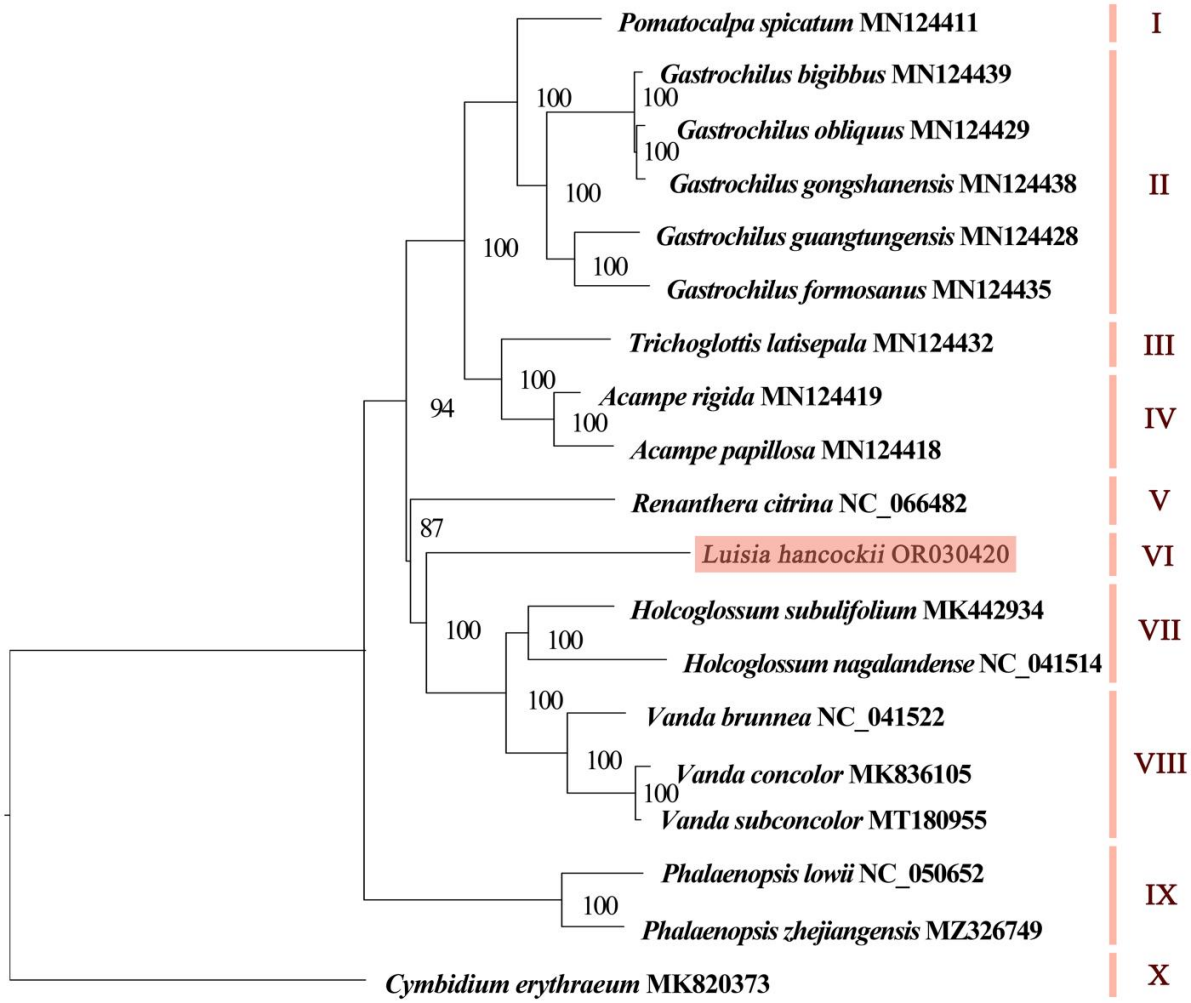
The maximum-likelihood tree based on complete chloroplast genome sequences of *Luisia hancockii* and 17 species of subtribe Aeridinae, with *Cymbidium erythraeum* as the outgroup species. The numbers next to the nodes indicate bootstrap support values. NCBI accession numbers of each genome are shown in the figure. The following sequences were used: *V. subconcolor* MT180955 (Liu, Tu, Zhang, et al. 2020), *Pomatocalpa spicatum* MN124411 (Liu, Tu, Zhao, et al. 2020), *Gastrochilus bigibbus* MN124439 (Liu, Tu, Zhao, et al. 2020), *G. obliquus* MN124429 (Liu, Tu, Zhao, et al. 2020), *G. gongshanensis* MN124438 (Liu, Tu, Zhao, et al. 2020), *G. guangtungensis* MN124428 (Liu, Tu, Zhao, et al. 2020), *G. formosanus* MN124435 (Liu, Tu, Zhao, et al. 2020), *Trichoglottis latisepala* MN124432 (Liu, Tu, Zhao, et al. 2020), *Acampe rigida* MN124419 (Liu, Tu, Zhao, et al. 2020), *A. papillosa* MN124418 (Liu, Tu, Zhao, et al. 2020), *Renanthera citrina* NC_066482 (Xiao et al. 2022), *Holcoglossum subulifolium* MK442934 (Li et al. 2019), *H. nagalandense* NC_041514 (Li et al. 2019), *Vanda brunnea* NC_041522 (Li et al. 2019), *V. concolor* MK836105 (Chen et al. 2019), *Phalaenopsis lowii* NC_050652 (Wang et al. 2019), *P. zhejiangensis* MZ326749 (Jiang et al. 2021), and *Cymbidium erythraeum* MK820373 (Huang et al. 2019).

The chloroplast genome consisted of 129 genes, consisting of 74 protein-coding genes, eight rRNAs, 38 tRNAs, and nine pseudogenes. Pseudogenes included seven *ndh* genes (*ndhG*, *ndhD*, *ndhE*, two copies of *ndhB*, *ndhJ*, and *ndhK*) and two copies of *ycf68*. Two small subunit of ribosome genes (*rpl2* and *rpl23*), three small subunit of ribosome genes (*rps7*, *rps12*, and *rps19*), four rRNA genes (*rrn4.5*, *rrn5*, *rrn16*, and rrn*23*), eight tRNA genes (*trnA-UGC*, *trnH-GUG*, *trnI-CAU*, *trnI-GAU*, *trnL-CAA*, *trnN-GUU*, *trnR-ACG*, and *trnV-GAC*), and two *ycf* genes (*ycf2* and *ycf68*) were comprised of two copies. Genes like *rps16*, *atpF*, *rpoC1*, *petB*, *petD*, *rpl16*, and *rpl2* contained one intron, while *ycf3* and *clpP* hosted two introns (Figure S2). The *rps12* is a trans-splicing gene (Figure S3). Phylogenetic tree based on complete chloroplast genomes revealed that 19 species could be divided into 10 groups, and the results were consistent with traditional morphology-based classification. *L. hancockii* clustered independently in group VI, and it was sister to *Holcoglossum* and *Vanda* species, with a support rate of 100% (Figure 3).

## Discussion and conclusions

To our knowledge, this is the first complete chloroplast genome sequence in the genus *Luisia*. In our study, seven *ndh* genes were identified as pseudogenes because of the presence of internal stop codons, while *ndhC* was lost in *L. hancockii* plastome. The pseudolization and loss of *ndh* genes in some orchid species like *P. equestris* (Schauer) Rchb. 1850, *Dendrobium officinale* Kimura & Migo 1936, *Goodyera fumata* Thwaites 1861, and *Masdevallia picturata* Rchb. 1878 are common (Lin et al. 2015, 2017). Moreover, two copies of *ycf68* genes were both pseudolized due to internal stop codons. Gene pseudolization in *ycf68* was also reported in *Utricularia reniformis* Saint-Hilaire 1830 and *Salix wilsonii* Seemen 1905 (Silva et al. 2016; Chen et al. 2019).

In conclusion, the *L. hancockii* chloroplast genome was assembled and annotated for the first time, and the genome was determined to be 146,243 bp in length, containing a total of 129 genes. The phylogenetic tree revealed *L. hancockii* is closely related to *Holcoglossum* and *Vanda* species. Our study will provide insights into genetic conservation and phylogenetic studies in Orchidaceae.

## Supplementary Material

Supplemental Material

## Data Availability

The data that support the findings of this study are openly available in GenBank of NCBI at https://www.ncbi.nlm.nih.gov/nuccore/OR030420. The associated BioProject, SRA, and Bio-Sample numbers are PRJNA974942, SRR24684304, and SAMN35302027, respectively.
