# Supplementary material for: Complete chloroplast genome of a traditional medicinal plant Luisia hancockii Rolfe 1896: genomic features and phylogenetic relationship within subtribe Aeridinae (Orchidaceae)
Source: Mitochondrial DNA B Resour. 2023 Oct 30;8(11):1149–53. doi: 10.1080/23802359.2023.2275334 (PMC10769519; doi:10.1080/23802359.2023.2275334)
Supplement: Supplemental Material [file TMDN_A_2275334_SM8137.pdf]

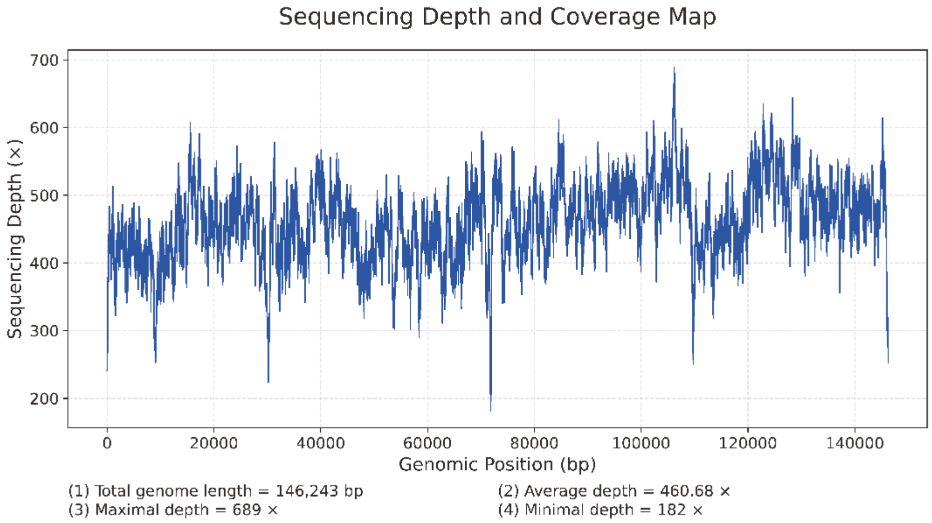

Figure S1 Coverage depth figure of the *Luisia hancockii* chloroplast genome. The horizontal coordinate is the position of the chloroplast genome, and the vertical coordinate is the sequencing depth.

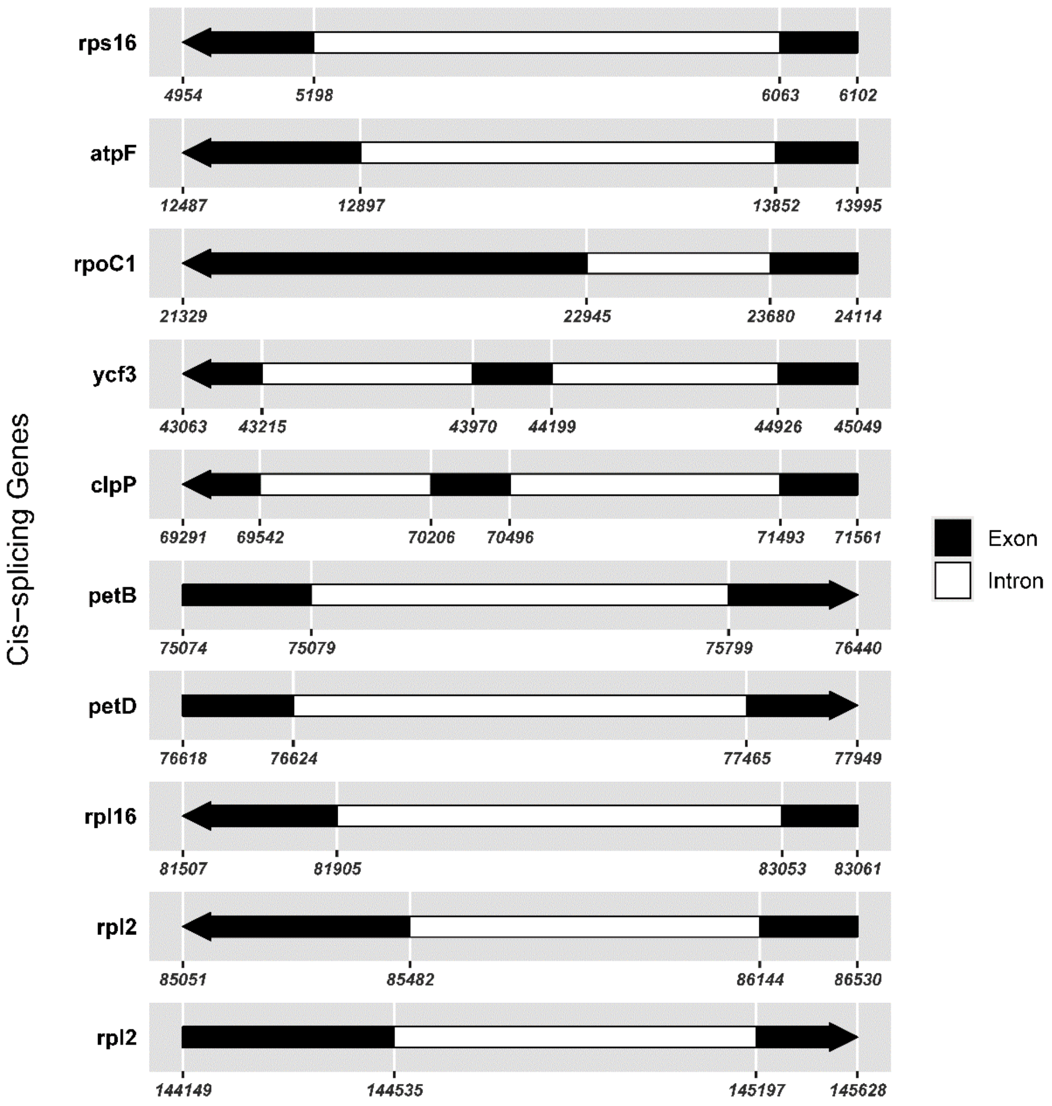

Figure S2 Schematic map of the cis-splicing genes in *Luisia hancockii* chloroplast genome.

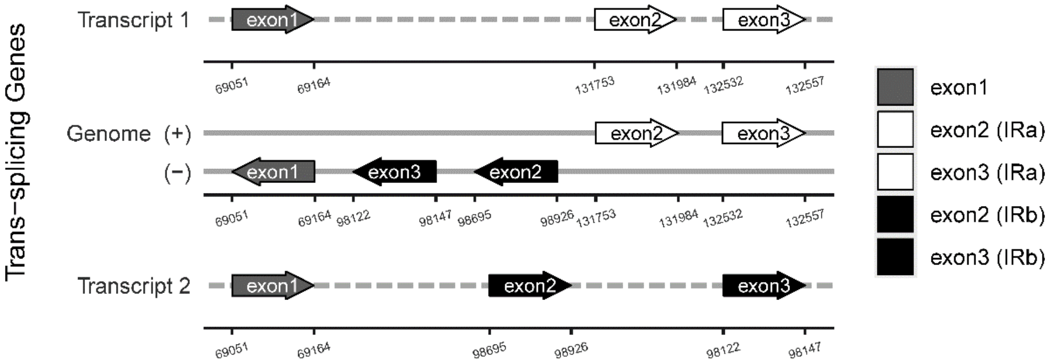

Figure S3 Schematic map of the trans-splicing gene *rps12* in *Luisia hancockii* chloroplast genome.
